# Hyperhemolysis in the Setting of Mixed-Autoimmune Hemolytic Anemia: A Rare Complication of COVID-19

**DOI:** 10.7759/cureus.20356

**Published:** 2021-12-12

**Authors:** Sonya Narula, Sean Winkle, Kenneth Brand, Farhan Shah, Priscilla Fujikawa, Tyler Truitt, Mark Currie

**Affiliations:** 1 Internal Medicine, LewisGale Medical Center, Salem, USA

**Keywords:** covid 19, autoimmune hemolytic anemia (aiha), hyperhemolysis syndrome, mixed autoimmune hemolytic anemia, immune thrombocytopenia purpura

## Abstract

Severe acute respiratory syndrome coronavirus 2 (SARS-CoV-2) is a novel human pathogen known for its predilection on the respiratory system. Herein, we present a unique case in which a patient developed hyperhemolysis in the setting of mixed autoimmune hemolytic anemia (AIHA) secondary to SARS-CoV-2. A 33-years-old male with a past medical history of resolved immune thrombocytopenic purpura (ITP) presented to the hospital with symptoms of jaundice after being infected with SARS-CoV-2. On admission, his Hgb was 12.5 g/dL. Lab results showed indirect bilirubin of 13 mg/dL, LDH at 759 U/L, haptoglobin <10, and the percent reticulocyte count was 2.33%. A direct antiglobulin test (DAT) was also positive for C3, IgG, anti-E, in addition to both warm and cold autoantibodies. PCR was positive for COVID-19. Within two days of admission, his Hgb dropped to 5.9 g/dL. A total of seven units of packed red blood cell (pRBC) was required to achieve a Hgb of 6 g/dL in 48 hours. Patients with preexisting hematological abnormalities have a propensity to develop AIHA in the setting of the virus. The majority of the cases described in the literature were associated with warm AIHA. Our patient tested positive for both warm and cold antibodies, which may partially explain the mechanism behind hyperhemolysis in our patient.

## Introduction

Severe acute respiratory syndrome coronavirus 2 (SARS-CoV-2) is an emerging human pathogen that is known to cause pneumonia, respiratory failure, and acute respiratory distress syndrome. While predominantly known for disease and complications involving the respiratory system, the pathology of the virus can cause sequelae to multiple organ systems [1]. To the best of our knowledge, there have been several cases of SARS-CoV-2-associated autoimmune hemolytic anemia (AIHA). Herein, we report a unique case of hyperhemolysis in the setting of the virus.

## Case presentation

A 33-year-old male with a past medical history of resolved immune thrombocytopenic purpura (ITP) presented to the hospital with acute onset “yellow eyes” and “orange urine” associated with a week of fevers, chills, and night sweats. This was associated with malaise, loss of taste and smell, cough, and shortness of breath. He presented to our hospital before the advent of SARS-CoV-2 vaccines.

On initial presentation the patient’s vital signs were normal. Labs were significant for bilirubin of 10 mg/dL; other liver enzymes were unremarkable. He had leukocytosis with a white blood cell count (WBC) of 15 × 103/µL, however, hemoglobin (Hgb) and platelet count (Plt) were normal. Polymerase chain reaction (PCR) for SARS-CoV-2 was negative. The only significant physical exam finding was scleral icterus.

Overnight, the patient reported night sweats, nausea, and non-bloody and non-bilious emesis. Indirect and total bilirubin had increased to 13 mg/dL and 14 mg/dL, respectively. His hemoglobin fell from 12.5 g/dL to 8.7 g/dL. He was started on IV dexamethasone 4 mg daily. A bone marrow biopsy was done due to concern for hemophagocytic lymphohistiocytosis, showing mild hemophagocytosis. An abdominal CT scan and a right upper quadrant ultrasound showed no hepatobiliary, pancreatic, or splenic involvement. Hemolysis labs results showed lactate dehydrogenase (LDH) at 759 U/L, Haptoglobin <10, ferritin 4903 ug/L, and the percent reticulocyte count was 2.33%.

Serologic testing performed for hepatitis, HIV, Echovirus, and cytomegalovirus were negative. The patient’s mycoplasma IgM returned as positive, though the titer was <770 U/L. A repeat direct antiglobulin test (DAT) was positive for IgG, C3d, and on further testing, he was found to have a cold autoantibody and anti-E. His blood sample was reactive with three out of three cells in the antibody screen, and this patient had a complement activating IgM, causing red blood cell (RBC) lysis. At this point, IgG for SARS-CoV-2 also came back positive. Hemolysis causing severe anemia (Hgb 4.7 g/dL) led to intensive care unit transfer one day after being admitted (Table 1). In addition to further packed red blood cell (pRBC) transfusion, intravenous immunoglobulin (IVIG) was given with steroids in the ICU as well. A total of seven units of pRBC was required to achieve a Hgb of 6.0 g/dL in 48 hours. Transfer to an outside facility was required for possible plasmapheresis.

**Table 1:** Laboratory values during days 1 and 3

	Reference range	Admission	Day 3
WBC (/µL)	4.50–10.5	15 × 10^3 ^	74 × 10^3^
HgB (g/dL)	11.4–15.5	12.5	4.7
Platelet (/µL)	130–385	344 × 10^9 ^	126 × 10^9 ^
Sodium (mmol/L)	135–145	136	136
Potassium (mmol/L)	3.6–5.2	4.4	4.6
Chloride (mmol/L)	100–108	105	105
Bicarbonate (mmol/L)	21–32	25	25
BUN (mg/dL)	7–18	21	32
Creatinine (mg/dL)	0.60	1.00	1.30
Glucose (mg/dL)	74–106	119	141
AST (U/L)	15–37	58	168
ALT (U/L)	13–61	51	67
Alkaline phosphatase (U/L)	45–117	67	86
Total bilirubin (mg/dL)	0.2	14	10.60
Direct bilirubin (mg/dL)		0.90	
LDH (U/L)	84–246	758	
Haptoglobin		<10	
Ferritin (ng/mL)	26–388	4903	
Reticulocyte count		2.3%	
Mycoplasma IgM		<770	
ESR		100 mm/hour	

At the outside facility, he was febrile to 39.1 °C, blood pressure was 134/74, heart rate 74, and respiratory rate of 35. His Hgb was 3.0 g/dL, WBC 74 × 10^3^/µL, and Plt 363 × 10^9^/L without evidence of overt bleeding. On the complete metabolic panel, his creatinine had risen to 1.34 mg/dL, AST of 303 µ/L, ALT 72 µ/L, and alkaline phosphatase 100 U/L, total bilirubin of 10.1 mg/dL, and conjugated bilirubin of 1.8 mg/dL. His reticulocyte count was 1.6%. He tested positive for SARS-CoV-2 via PCR. The patient was started on rituximab in addition to IVIG and methylprednisone. He received 1U pRBC, but he rapidly deteriorated and developed multiorgan failure and coagulopathy with disseminated intravascular coagulation. His electrocardiogram showed anterolateral and inferior ischemia. He subsequently developed cardiac arrest. Massive transfusion protocol was initiated, and he received 4U pRBC. However, he ultimately died from shock secondary to a hyperhemolytic state.

## Discussion

AIHA following SARS-CoV-2 infection with either warm or cold autoantibodies has previously been documented in the literature [2]. The majority of the cases described in the literature were associated with warm AIHA [3]. To the best of our knowledge, this has been the first case in which a patient had tested positive for both warm and cold antibodies. Previously reported median time between the onset of SARS-CoV-2 and symptoms of AIHA was nine days, and the range was anywhere between 4 and 13 days [2]. Our patient presented to our hospital 14 days after his initial symptom of cough. Some of the reported cases of SARS-CoV-2 associated AIHA had a history of a prior hematological disorder. This suggests that preexisting hematological abnormalities may be a risk factor in the development of AIHA. These patients were treated with a combination of steroids and/or IVIG with the addition of rituximab in refractory cases [2]. 

This patient presented with an unusual complication known as hyperhemolysis. Hyperhemolysis is a type of delayed hemolytic transfusion reaction in which there is both hemolysis of transfused RBCs as well as the recipient’s RBCs. Though originally described in AIHA, it has most commonly been observed in sickle-cell disease (SCD) patients who have required multiple transfusions [4]. Since hyperhemolysis is typically seen with complement activation of the membrane attack complex, which is uncommon in IgG predominant AIHA, it is likely that the patient’s presentation with mixed warm-cold antibodies triggered his pathology. 

SARS-CoV-2 may trigger AIHA through molecular mimicry, in which antibodies attack self-antigens due to shared sequences between foreign antigens and self-antigens. Ankyrin-1 is an erythrocyte membrane protein whose main function is to provide a connection between the membrane skeleton and plasma membrane. This protein shares a putative immunogenic antigenic epitope with 100% identity with a SARS-CoV-2 surface glycoprotein known as spike protein [5]. In addition to this, it has been postulated that a modification of the surface of the RBC occurs during the course of the infection and hyperinflammation may further enhance complement deposition [6]. This alteration of erythrocyte membrane protein may influence the degree of hemolysis in SARS-CoV-2 patients, however, to the best of our knowledge this has been only shown in vitro. Whether having some underlying hematological/oncological abnormality plays a role in the ability of the virus to modify the surface of the RBCs is yet to be determined.

Researchers have posited that systemic hyperinflammation leading to a cytokine storm is the underlying mechanism behind the mortality of SARS-CoV-2. Specifically, serum cytokine levels that have been found to be elevated in patients suffering from a SARS-CoV-2-associated cytokine storm include interleukin-1β, interleukin-6, inducible protein-10, tumor necrosis factor, interferon-γ, macrophage inflammatory protein 1α and 1β, and vascular endothelial growth factor. Also, increased interleukin-6 (IL-6) levels have been strongly associated with a shorter survival amongst patients suffering from SARS-CoV-2 [7]. The use of IL-6 inhibitors like tocilizumab has been considered since researchers have noted high concentrations of proinflammatory cytokines, such as IL-6 in severe SARS-CoV-2 infections [8]. It has also been shown to be effective in patients who develop hyperhemolysis in the setting of SCD [9].

## Conclusions

Of the cases of AIHA described in the literature, this is the first case of a patient who developed hyperhemolysis in the setting of mixed AIHA secondary to SARS-CoV-2 outside of SCD. Our patient tested positive for both warm and cold antibodies, which may partially explain the mechanism behind hyperhemolysis. This case highlights the importance of further monitoring of SARS-CoV-2 infections and study of the pathophysiology associated with extrapulmonary manifestations of the virus. As a better understanding of associated systemic disease is reported, practitioners will be more prepared to anticipate poor outcomes and treat patients accordingly.
